# Diet Quality of Australian Children and Adolescents on Weekdays versus Weekend Days: A Secondary Analysis of the National Nutrition and Physical Activity Survey 2011–2012

**DOI:** 10.3390/nu13114128

**Published:** 2021-11-17

**Authors:** Dimity C. Dutch, Rebecca K. Golley, Brittany J. Johnson

**Affiliations:** Caring Futures Institute, College of Nursing and Health Sciences, Flinders University, Bedford Park, SA 5042, Australia; dimity.dutch@flinders.edu.au (D.C.D.); rebecca.golley@flinders.edu.au (R.K.G.)

**Keywords:** discretionary choices, energy-dense nutrient-poor foods, diet quality, children, adolescents, weekday, weekend

## Abstract

Daily routines may influence children and adolescents’ eating patterns, however the influence of days of the week on dietary intake has rarely been explored. This study aimed to examine discretionary choices intake in the context of diet quality on weekdays versus weekends. A secondary analysis was conducted using the Australian National Nutrition and Physical Activity Survey 2011–2012. Differences in discretionary choices intake and diet quality on weekdays versus weekends, were examined using ANCOVA analyses. Associations between child and parent-proxy characteristics and weekday/weekend discretionary choices intake were examined using multivariable regression models. Primary analyses included 2584 Australian 2–17-year-olds. There were small differences in discretionary choices intake and diet quality between weekdays and weekends in all age subgroups. Compared to weekdays, intakes on weekends were characterized by a higher intake of discretionary choices, and lower total Dietary Guidelines Index for Children and Adolescents (DGI-CA) scores across the age subgroups (all *p* < 0.01). Parent-proxy discretionary choices intake and child age were predictors of weekday and weekend discretionary choices intake. Parent-proxy obesity weight status compared with healthy weight status was a predictor of weekend intake, while parent-proxy education level was a predictor of weekday discretionary choices intake. Future intervention strategies should target discretionary choices intake on both weekdays and weekends.

## 1. Introduction

Many health conditions are associated with poor diet quality and an excessive intake of energy-dense, nutrient-poor foods, termed discretionary choices in the Australian Dietary Guidelines [1,2,3,4]. Consumption of discretionary choices may also displace the intake of nutrient-dense foods and indirectly contribute to poorer dietary quality [4]. In Australia, discretionary choices contribute to 39% of total energy, 49% of saturated fat, 87% of added sugars and 51% of sodium intake, and less than 1% of children and adolescents aged 2–18 years meet recommended serves of vegetables [5,6,7]. Similar intakes have been identified internationally in the United States [8], Mexico [9] and Switzerland [10].

As there has been little improvement in the dietary intake and patterns of children and adolescents over recent decades [11], new insights and approaches are needed. There are many interrelated factors that affect dietary intake including social, psychological, environmental, cultural and economic influences [12,13,14]. Daily routines, including the day of the week, is an under-examined influence that may be particularly relevant to the intake of discretionary choices.

The social and cultural differences between weekday and weekend routines and the impact on dietary intake needs further exploration [15]. A review of 190 studies investigating health behaviors in children found consistent evidence regarding the “Structured Days Hypothesis” and the positive influence that the structured work or school routine of weekdays has on health behaviors [16]. The role of the school environment and influence of peers are also key factors to consider when examining weekday dietary intake. Qualitative literature suggests that parents feeding practices change depending on the day of the week. For example, the restriction of discretionary foods is more common on weekdays [17,18]. On the contrary, weekend days are typically periods of less structure and include more social occasions. Weekends are also related to an increase in access to food within the home and greater consumption of meals outside of home, both associated with poorer diet quality and higher energy intake [19].

Weekday–weekend differences in dietary intake have been investigated in the United States, Denmark, Canada and Australia within adult and/or pediatric populations with findings of a less healthful diet on weekends compared to weekdays [20,21,22,23,24]. A 2007 Australian study of 6 to 16-year-olds compared dietary intake on school versus non-school days. However, predictors of discretionary choices intake were not investigated [21]. Contradictory findings have been reported in American [25] and New Zealand [26] populations. Limitations in these studies include selection bias and not adjusting for confounders.

The present analysis aimed to (1) examine discretionary choices intake in the context of diet quality comparing weekdays versus weekend days and (2) identify and compare predictors of discretionary choices intake on weekdays versus weekend days in a contemporary representative sample of Australian children and adolescents aged 2 to 17 years. It was hypothesized that discretionary choices intake would be higher on weekends, whereas diet quality would be lower on weekends compared to weekdays. The current analysis will help to inform potential intervention targets and strategies for improving dietary intake in line with national guidelines.

## 2. Materials and Methods

### 2.1. Study Design and Participants

This study was a secondary analysis of the 2011–2012 National Nutrition and Physical Activity Survey (NNPAS), a cross-sectional survey conducted by the Australian Bureau of Statistics between May 2011 and June 2012, which, however, remains the most contemporary nationally representative comprehensive data available. A full description of survey scope and methodology has been reported previously by the Australian Bureau of Statistics [27]. Briefly, the sample was selected using a stratified, multistage, area sample of private dwellings. Children and adolescents were invited to take part in the national survey following the recruitment of an adult within the household resulting in 12,153 individuals aged 2 years and over included in the final sample. This analysis used data from the 2584 children and adolescents aged 2 to 17 years and the corresponding 2584 adults from the same households. Child–adult dyads were identified according to household identification number (*n* = 2584) and represent parent-proxy in the current analysis. Although relationship to the child was not reported in the data set, adults within the same household are referred to as parent-proxy throughout.

### 2.2. Dietary Assessment

Dietary intake data was collected by trained and experienced interviewers on two occasions via a 24-h dietary recall using the Automated Multiple-Pass Method on a computer-assisted interview system [28,29]. The 5-pass method, originally developed by the United States Department of Agriculture, was used to ensure complete and consistent dietary data collection [28]. Dietary information was collected via a proxy interviewer (i.e., parent or adult) with or without assistance from the child. Only day one dietary intake information collected during the face-to-face computer-assisted personal interview was used in this analysis, as only 64% of respondents participated in the second dietary recall. Food model booklets and household measures were also utilized to assist respondents in describing portion sizes of food and beverages consumed within the previous 24 h. As weekday and weekend intake data was not available from the same child, the weekday and weekend samples in this study describe different children.

### 2.3. Dietary Intake

Dietary outcome measures used in this analysis include discretionary choices intake presented as ”serves” and percent of total energy (%E), total saturated fat (g/day), added sugars (g/day), sodium (mg/day) and fiber intake (g/day). One “serve” of discretionary choices is equivalent to 600 kJ (143.4 kcal) [4]. Discretionary choices as percent of total energy intake (%E) was used as the main outcome variable to allow comparisons across age groups. Nutrients from all food and beverages reported were estimated using the Food Standards Australia New Zealand AUSNUT 2011–2013 database, a food composition database developed specifically for the 2011–2012 NNPAS [30]. Each food and beverage within the database is flagged as either discretionary or non-discretionary based on the definition according to the Australian Dietary Guidelines [30]. Although there are other classifications for these types of foods, such as the NOVA Food Classification System [31], the discretionary flag was used to align with the Australian Dietary Guidelines definition. Diet quality was assessed using the Dietary Guidelines Index for Children and Adolescents (DGI-CA), which provides a measure of Australian children and adolescents’ adherence to the Australian Dietary Guidelines [1]. The index incorporates 11 indicators that reflect concepts of diet variety, adequacy, quality and moderation and provides a score from 0–100 with a higher score reflecting greater adherence to the Australian Dietary Guidelines. Further details of the DGI-CA have been reported elsewhere [1,32]. A DGI-CA total score was calculated for each child and adolescent within the dataset. Although differing definitions for weekday versus weekends exist in the literature, for the current analyses weekdays were defined as Monday to Thursday, and weekends defined as Friday to Sunday.

### 2.4. Socio-Demographic Characteristics and Covariates

Child and adolescent characteristics included in this analysis include age, sex, weight status and adherence to physical activity and sedentary behavior recommendations. Adherence to activity recommendations was expressed as the number of days children and adolescents met age-specific recommendations for physical and screen-based activity. Parent-proxy characteristics include weight status, sex and discretionary choices intake (%E). Multiple indicators of socio-economic position were also used including parent-proxy education level (tertiary, diploma/certificate, high school or less), parent-proxy employment status (employed, unemployed, not in labor force), equivalized household income deciles and Socio-Economic Indexes for Areas quintiles.

Body weight and height were measured by a trained assessor during the personal interview using digital scales and a stadiometer, respectively, and were used to calculate body mass index (BMI). Participants were then grouped into weight status categories (underweight, healthy weight, overweight and obesity) according to the World Health Organisation international classification for adults, and corresponding age- and sex-specific cut-off values for children and adolescents [33,34].

### 2.5. Statistical Analysis

All statistical analyses were performed using the statistical software package IBM SPSS Statistics (Version 25; SPSS Inc., Chicago, IL, USA). The level of significance was set at *p* < 0.01 for all analyses. Differences between demographic characteristics of weekday and weekend samples were tested using a chi square for independence for categorical variables (sex, BMI, education level, employment status and Socio-Economic Indexes for Areas). Variables treated as continuous were assessed for normality, with independent samples *t*-test or Mann–Whitney U test conducted, as appropriate, for age, number of days met physical activity and screen-based recommendations and household income deciles.

Differences in intake on weekday and weekends were examined by child age subgroups based on the school system, with 2- to 4-year-olds defined as preschool, 5- to 11-year-olds as primary school and 12- to 17-year-olds as secondary school. Mean intakes of discretionary choices, associated key nutrients, dietary fiber and total DGI-CA score were compared between weekdays and weekends using ANCOVA analyses, adjusting for differences between weekday and weekend samples. Data are presented as estimated marginal means and standard error.

Multivariable linear regression models were used to determine the association between child and parent-proxy socio-demographic variables and child discretionary choices intake (%E) on weekday versus weekend days. Regressions used the combined sample of 2- to 17-year-olds. Assumptions of regression analysis were tested by checking the normality, linearity and homoscedasticity of residuals [35]. Differences between the two sample groups were included as covariates (parent-proxy sex and child physical activity and sedentary behavior). Predictors in the regression included child age, child sex, child weight status, parent-proxy weight status, parent-proxy education level, parent-proxy employment status, household income decile and parent-proxy discretionary choices intake (%E). Categorical variables were dummy coded. Unstandardised regression coefficients (B), standard error and standardized coefficients (Beta) were used to evaluate the strength of associations. The sample size of multivariable regression analyses (Weekday *n* = 1540 and Weekend *n* = 777) met the required sample size of *n* = 210 as 14 predictor variables were included in the regressions and recommendations indicate 15 participants per predictor variable, and the sample size was also deemed suitable for ANCOVA analyses [35].

### 2.6. Sensitivity Analysis

Two sensitivity analyses were performed to assess any differences in results when considering only plausible reporters or using a different definition of weekday (Monday to Friday) and weekends (Saturday and Sunday). Plausible reporters were determined using Goldberg cut-off values, excluding both under- (EI:BMR ratio of <0.87) and over-reporters (EI:BMR > 2.74), by comparing energy intake (EI) to basal metabolic rate (BMR) ratio with energy expenditure (PAL, of 1.55) [36,37]. Individuals with missing data for EI:BMR (*n* = 450) were not considered plausible reporters [27], as it was not possible to asses if their intake was plausible. The first sensitivity analysis included a total sample of 1826 (weekday *n* = 1200; weekend *n* = 626) plausible reporters, 71% of the total analysis sample.

## 3. Results

### 3.1. Study Population

Table 1 summarizes the characteristics of the weekday and weekend samples of children and adolescents aged 2 to 17 years included in the current analyses (*n* = 2584 child–adult dyads). The average age of the children and adolescents included in the weekday sample was 9.0 ± 4.9 years, half (50%) were female and approximately 27% were classified as being overweight or with obesity. The corresponding parent-proxy sample had an average age of 39.7 ± 9.9 years, approximately 61% were female, 61% were classified as overweight or with obesity and 65% had attained an education level greater than high school. The weekend samples were similar, with children and adolescents having an average age of 9.4 ± 4.9 years, just under half (48%) were female and approximately 26% were classified as being overweight or with obesity. The average age of the weekend parent-proxy sample was 39.8 ± 8.7 years, 54% were female, 62% were classified as overweight or with obesity and 69% had attained an education level greater than high school. Sixty-seven percent of participants reported dietary intake on a weekday (Monday–Thursday). Supplementary Table S1 shows the characteristics of the age subgroups. There were no significant differences between weekday and weekend samples in the preschool and secondary school age groups. Characteristics for primary school aged weekday and weekend samples were similar except for parent-proxy employment status and equivalized household income.

### 3.2. Dietary Intake

#### 3.2.1. Preschool

Table 2 shows the mean discretionary choices intake, associated nutrient intake and total DGI-CA score on weekdays (*n* = 426) and weekend (*n* = 186) days of preschool aged children (2–4 years). Intake of discretionary choices was higher on weekend days compared to weekdays in both serves (mean difference 0.6 serves; *p* < 0.001) and percent of energy intake (mean difference 5.1%E; *p* < 0.001). Saturated fat intake (mean difference 1.1 g; *p* = 0.009) was significantly higher on weekend days, compared to weekdays. Total DGI-CA score (mean difference −3.35 points; *p* < 0.001) was lower on weekend days, compared to weekdays.

#### 3.2.2. Primary School

Table 3 presents the mean discretionary choices intake, associated nutrient intake and total DGI-CA score on weekdays (*n* = 627) and weekend days (*n* = 309) in primary school aged children (5–11 years). Intake of discretionary choices was higher on weekend days, compared to weekdays in serves (mean difference 1.0 serves; *p* = 0.003), but was not statistically significant for discretionary choices as a percent of energy intake (mean difference 3.9%E; *p* = 0.012). Sodium intake (mean difference 254 mg; *p* = 0.001) was significantly higher on weekend days, compared to weekdays. Total DGI-CA score (mean difference −1.6 points; *p* = 0.006) was lower on weekend days, compared to weekdays.

#### 3.2.3. Secondary School

Table 4 shows mean discretionary choices intake, associated nutrient intake and total DGI-CA score on weekdays (*n* = 522) and weekend days (*n* = 292) in adolescents (12–17 years). Intake of discretionary choices was higher on weekend days compared to weekdays in both serves (mean difference 0.9 serves; *p* = 0.073) and percent of energy intake (mean difference 3.3%E; *p* = 0.129), however, results were not statistically significant. Added sugars intake (mean difference 16.5 g; *p* = 0.001) was significantly higher on weekend days compared to weekdays. Fiber intake (mean difference −2.2 g; *p* = 0.004) and total DGI-CA score (mean difference −3.24 points; *p* = 0.001) were lower on weekend days compared to weekdays.

### 3.3. Predictors of Discretionary Choices Intake

Table 5 shows the results of the multivariable linear regression analyses used to identify predictors of children and adolescents’ weekday (*n* = 1540) and weekend (*n* = 777) discretionary choices intake (%E) after adjusting for potential confounders. Parent-proxy obesity status compared to healthy weight was significantly associated with higher weekend discretionary choices intake (B = 5.307; SE 1.842; Beta = 0.110; *p* = 0.004). Lower parent-proxy education level, when compared to tertiary attainment, was a significant predictor for weekday discretionary choices intake (B = 4.387; SE 1.247; Beta = 0.105; *p* < 0.001). Parent-proxy discretionary choices intake (weekday B = 0.302; SE 0.023; Beta = 0.318; *p* < 0.001, weekend B = 0.282; SE 0.034; Beta = 0.289; *p* < 0.001) and child age (weekday B = 0.785; SE 0.108; Beta = 0.187; *p* < 0.001, weekend B = 0.465; SE 0.161; Beta = 0.107; *p* = 0.004) were significant predictors of both higher weekday and higher weekend discretionary choices intake in children and adolescents.

### 3.4. Sensitivity Analyses

There were no substantial differences within the preschool age sample when examining differences in weekday and weekend samples in only plausible reporters or differing definition of weekday/weekends (data available upon request). Sensitivity analysis in the primary and secondary age samples including only plausible reporters showed similar differences between weekday and weekend samples. Within the primary school sample, discretionary choices intake was higher on weekend days in both %E (mean difference 4.4%E; *p* = 0.008) and serves (mean difference 0.9 serves; *p* = 0.002), as opposed to only serves in the primary analysis. In the secondary school age sample, fiber was no longer found to be significantly lower on weekend days (mean difference −2.4 g; *p* = 0.019). In the sensitivity analysis, using a different definition of weekday/weekends in the primary school and secondary school age sample found similar patterns of results with the exception of serves of discretionary choices (5–11 yo mean difference 0.9 serves; *p* = 0.028) and added sugar (12–17 yo mean difference 12.5 g; *p* = 0.031) were not significantly different when comparing weekday versus weekend. There were no differences in the key predictors of weekday or weekend discretionary choices intake in the sensitivity analysis of plausible reporters (data available upon request). The sensitivity analysis by different definition of weekday/weekend found no difference in predictors of weekday discretionary choices intake. For the weekend sample, parent-proxy discretionary choices intake (Beta = 0.319; *p* < 0.001), parent-proxy overweight status (Beta = 0.136; *p* = 0.005) and parent-proxy obesity status (Beta = 0.148; *p* = 0.002) were found to be predictors of child discretionary choices intake (%E) on weekends.

## 4. Discussion

Our study examined children and adolescents’ discretionary choices intake, in the context of diet quality on weekends versus weekdays within age subgroups and explored predictors of weekday and weekend discretionary choices intake. We found small differences in discretionary choices intake and indicators of diet quality between weekdays and weekend days in all age subgroups within a nationally representative sample of Australian children and adolescents. Weekend dietary intakes were characterized by a higher intake of discretionary choices, and a lower total DGI-CA score was generally consistent across the age subgroups compared to weekdays. However, overall, both weekday and weekend intakes were characterized by high intakes of discretionary choices. Predictors of discretionary choices intake identified in this analysis were parent-proxy obesity status for weekend days, parent-proxy education level for weekdays and parent-proxy discretionary choices intake and child age for both weekdays and weekend days.

The current analysis indicates suboptimal dietary intake regardless of the day of the week, despite likely differences in routines and eating environments. Previous studies in the U.S. [22], New Zealand [26] and Denmark [23], despite methodological differences, have reported consistent findings of a less healthful diet and higher energy intake, ranging from an additional 120 kJ [22] to 1300 kJ [23] on weekends compared to weekdays in children and adolescents (2–18 yo, 4–14 yo, 5–14 yo, respectively). Similar to our results, Grimes et al. [21] found intakes of Australian school children aged 6 to 16 years to be of poorer nutritional quality, including a greater consumption of discretionary choices (primary school: 32%, *p* < 0.001; secondary school: 30%, *p* < 0.001) and slightly higher intakes of sodium and saturated fat on non-school days (defined as Saturday and Sunday). A lack of consistency within the literature regarding how weekdays and weekend days are defined makes it difficult to understand the influence day of week has on dietary intake and quality. The differences between weekday versus weekend were smaller than anticipated. For example, the difference in discretionary choices serves across age subgroups ranging from 0.6 to 1.0 serves, compared with average daily consumption of 2.8 serves on weekdays in the preschool sample and 6.6 on weekends in the secondary school sample. The social and cultural differences between weekday and weekend routines are important to consider when investigating differences in dietary intake. During weekdays, children spend 6–7 h in school daily, with a previous study using the same national sample finding that within school hours, primary school aged children consume 37% of their daily energy intake, with 44% of that energy intake being from discretionary choices [38]. Children and adolescents’ dietary intake requires attention on both weekday and weekend settings.

In the present study, parent-proxy discretionary choices intake and child age were both predictors of weekday and weekend child discretionary choices intake. This is unsurprising due to the previously well-established bidirectional relationship between child and parent intake as parents are gatekeepers and role models within the home [39,40,41,42]. The broader literature has also reported the decline in diet quality throughout childhood and adolescence, related to increased autonomy, reduced parental influence and the increased influence of the school environment and the influence of peers [1,40,43,44]. To our knowledge, the previous literature has only investigated influences upon a child’s total intake, with no distinction of weekday predictors compared to weekend diet predictors. The lack of prior literature limits our ability to discuss the findings of parent-proxy education level and parent-proxy obesity status as predictors of weekday and weekend discretionary intake, respectively.

The current study is not without limitations. Study outcomes are based on self-reported dietary intake data, therefore, the consumption of discretionary choices reported may underestimate a potential larger true difference between weekday and weekend intake. We used single day intake, which is not representative of an individual’s usual intake. Weekday and weekend intake data were not available from the same child, however differences between groups were adjusted for in analyses to reduce any potential bias. Potential misclassification of a weekday could have also occurred as dates for school terms and holidays were not considered. There were also several strengths, including use of the most contemporary nationally representative data available at the time of analysis of Australian children and adolescents’ intake. Use of the Dietary Guideline Index for Children and Adolescence provided a comprehensive measure of diet quality that has also been associated with key nutrient intake and health-related outcomes [1]. Sensitivity analyses were conducted to determine if results changed using different weekday and weekend definitions. Misreporting of dietary intake was also considered by conducting further sensitivity analysis excluding both under- and over-reporters based on the Goldberg cut-off method to reduce the effects of measurement bias [36].

Overall, the findings from this study indicate the need to support and promote healthy eating behaviors every day of the week. Health promotion and intervention strategies need to focus on both parent and child due to the significant influence parents have on their child’s intake and the potential bidirectional nature of this relationship. The differences in intake identified in the current analysis may be reflective of discretionary choices displacing healthy foods within the diet, further contributing to poor diet quality. Future exploration of food choices in different contexts through qualitative research and examination of dietary intake behaviors and the social and environmental differences of weekdays versus weekend days, such as eating locations, food environments, time of day and eating occasions, could also assist in developing more targeted approaches and specific public health messages to improving dietary intake.

## 5. Conclusions

Australian children and adolescents have poor diet quality and high intakes of discretionary choices on both weekdays and weekend days. This study indicates that while weekend diets appeared to be slightly less healthful than weekdays, overall intake on both weekdays and weekend days is not in line with national dietary guidelines recommendations. Future health promotion strategies and interventions should target discretionary choices intake on both weekdays and weekend days, however, further qualitative research is required to explore barriers and enablers to making healthful diet choices at different times of the week to ensure strategies are appropriate for the social and cultural differences between weekdays and weekend days.

## Figures and Tables

**Table 1:** Characteristics of weekday and weekend samples of children and adolescents and parent-proxy from the National Nutrition and Physical Activity Survey 2011–2012 ^1^.

	Weekday (*Monday–Thursday*)		Weekend (*Friday–Sunday*)		*p*-Value ^6^
	*n*	%	*n*	%	
***Child Characteristics (2–17 yo)***	*n* = 1726		*n* = 858		
**Sex** (*n* = 2584)					0.309
Male	855	49.5	444	51.7	
Female	871	50.5	414	48.3	
**Age (years), mean (SD)** (*n* = 2584)	9.0	4.9	9.4	4.8	0.075
**BMI (kg/m^2^) ^2^** (*n* = 2122)					0.454
Underweight (<18.5)	75	5.4	32	4.4	
Healthy weight (18.5–24.99)	947	68.0	508	69.7	
Overweight (25–29.99)	257	18.4	140	19.2	
Obesity (>30)	114	8.2	49	6.7	
**Days met physical activity and screen-based recommendations, mean (SD) ^3^** (*n* = 2534)	3.1	2.5	3.2	2.4	0.280
***Parent-proxy Characteristics (18+ yo)***	*n* = 1726		*n* = 858		
**Sex** (*n* = 2584)					**0.001**
Male	672	38.9	392	45.7	
Female	1054	61.1	466	54.3	
**Age (years), mean (SD)** (*n* = 2584)	39.7	9.9	39.8	8.7	0.715
**BMI (kg/m^2^) ^2^** (*n* = 2215)					0.797
Underweight (<18.5)	19	1.3	10	1.3	
Healthy weight (18.5–24.99)	547	31.8	280	36.4	
Overweight (25–29.99)	479	33.1	271	35.2	
Obesity (>30)	401	27.7	208	27.0	
**Education Level** (*n* = 2584)					0.133
Tertiary	488	28.3	268	31.2	
Diploma/Certificate	632	36.6	320	37.3	
High school or less	606	35.1	270	31.5	
**Employment Status** (*n* = 2584)					**<0.001**
Employed	1279	74.2	700	81.6	
Unemployed	49	2.8	17	2.0	
Not in Labor Force	398	23.1	141	16.4	
**Household Income (deciles) ^4^, mean (SD)** (*n* = 2362)	5.3	2.7	5.8	2.7	**<0.001**
**SEIFA ^5^** (*n* = 2584)					0.128
Lowest quintile	305	17.7	134	15.6	
Second quintile	317	18.4	158	18.4	
Third quintile	369	21.4	158	18.4	
Fourth quintile	319	18.5	174	20.3	
Highest quintile	416	24.1	234	27.3	

^1^ All results presented as N (%) unless reported otherwise. ^2^ BMI, Body mass index categories; Underweight <18.5 kg/m^2^; Normal weight 18.5–24.99 kg/m^2^; Overweight 25–29.99 kg/m^2^; Obesity >30 kg/m^2^; missing data *n* = 333 weekday sample children, *n* = 129 weekend sample children, *n* = 280 weekday sample parent-proxy, *n* = 89 weekend sample parent-proxy. ^3^ Number of days met physical activity and screen-based recommendations in 7 days prior to interview, missing data *n* = 40 weekday sample children, *n* = 10 weekend sample children. ^4^ Gross weekly equivalized cash income of household; 1st decile <AUD 333, 2nd decile AUD 333–398, 3rd decile AUD 399–502, 4th decile AUD 503-638, 5th decile AUD 639–795, 6th decile AUD 796–958, 7th decile AUD 959–1151, 8th decile AUD 1152–1437, 9th decile AUD 1438–1917, 10th decile >AUD 1918, missing data *n* = 151 weekday sample, *n* = 71 weekend sample. ^5^ SEIFA, Socio-Economic Indexes for Areas Index of Relative Socio-Economic Disadvantage 2011—low index score indicated relatively greater disadvantage, high index score indicated a relative lack of disadvantage. ^6^ Differences between weekday and weekend samples tested using chi square for categorical variables (sex, BMI, education level, employment status and SEIFA) and independent samples *t*-test for continuous variables (age, physical activity and screen-based recommendations, household income deciles).

**Table 2:** Dietary intake of Australian preschool children (2–4 yo) on weekdays (Monday–Thursday) versus weekend days (Friday–Sunday).

	Weekday (*Monday–Thursday*) *n* = 426		Weekend (*Friday–Sunday*) *n* = 186		*p*-Value ^1^
	Mean	SE	Mean	SE	
Discretionary choices intake (%E)	27.1	0.9	32.2	1.4	**<0.001**
Discretionary choices intake (serves) ^2^	2.8	0.1	3.4	0.2	**<0.001**
Energy intake (kJ/day)	6012	97	6048	146	0.122
Saturated fat (g/day)	21.9	0.5	23.0	0.8	**0.009**
Added sugars (g/day)	32.8	1.4	35.7	2.2	0.079
Sodium (mg/day)	1625	35	1578	54	0.623
Fiber (g/day)	16.6	0.4	15.3	0.6	0.188
DGI-CA score (points/100) ^3^	47.0	0.6	43.7	0.9	**<0.001**

^1^ *p*-values from ANCOVA adjusted for parent-proxy employment status and parent-proxy equivalized income. ^2^ One serve of discretionary is 600 kJ. ^3^ DGI-CA, Dietary Guideline Index for Children and Adolescents [1].

**Table 3:** Dietary intake of Australian primary school children (5–11 yo) on weekdays (Monday–Thursday) versus weekend days (Friday–Sunday).

	Weekday (*Monday–Thursday*) *n* = 627		Weekend (*Friday–Sunday*) *n* = 309		*p*-Value ^1^
	Mean	SE	Mean	SE	
Discretionary choices intake (%E)	36.5	0.8	40.4	1.1	**0.012**
Discretionary choices intake (serves) ^2^	4.7	0.1	5.7	0.2	**0.003**
Energy intake (kJ/day)	7544	110	8098	157	0.039
Saturated fat (g/day)	26.8	0.6	30.0	0.8	0.013
Added sugars (g/day)	51.5	1.7	60.2	2.5	0.020
Sodium (mg/day)	2151	43	2405	62	**0.001**
Fiber (g/day)	20.2	0.4	19.7	0.5	0.250
DGI-CA score (points/100) ^3^	42.7	0.5	41.1	0.7	**0.006**

^1^ *p*-values from ANCOVA adjusted for parent-proxy employment status and parent-proxy equivalized income. ^2^ One serve of discretionary is 600 kJ. ^3^ DGI-CA, Dietary Guideline Index for Children and Adolescents [1].

**Table 4:** Dietary intake of Australian secondary school children (12–17 yo) on weekdays (Monday–Thursday) versus weekend days (Friday–Sunday).

	Weekday (*Monday–Thursday*) *n* = 522		Weekend (*Friday–Sunday*) *n* = 292		*p*-Value ^1^
	Mean	SE	Mean	SE	
Discretionary choices intake (%E)	37.6	0.9	40.9	1.2	0.129
Discretionary choices intake (serves) ^2^	5.7	0.2	6.6	0.7	0.073
Energy intake (kJ/day)	8972	161	9083	216	0.258
Saturated fat (g/day)	31.4	0.8	32.9	1.1	0.350
Added sugars (g/day)	65.8	2.4	82.3	3.3	**0.001**
Sodium (mg/day)	2640	62	2723	83	0.093
Fiber (g/day)	21.8	0.5	19.6	0.6	**0.004**
DGI-CA score (points/100) ^3^	38.7	0.65	35.4	0.7	**0.001**

^1^ *p*-values from ANCOVA adjusted for parent-proxy employment status and parent-proxy equivalized income. ^2^ One serve of discretionary is 600 kJ ^3^ DGI-CA, Dietary Guideline Index for Children and Adolescents [1].

**Table 5:** Multivariable associations between child and parent-proxy characteristics and discretionary choices intake on weekdays (Monday–Thursday) and weekend days (Friday–Sunday).

	Weekday ^2^ *n* = 1540 (*Monday–Thursday*)				Weekend ^2^ *n* = 777 (*Friday–Sunday*)			
	Unstandardized Regression Coefficient		Standardized Coefficients	*p*-Value ^3^	Unstandardized Regression Coefficient		Standardized Coefficients	*p*-Value ^3^
	*B*	Standard Error	*Beta*		*B*	Standard Error	*Beta*	
**Child sex** (*Male*)	0.912	0.948	0.023	0.336	0.940	1.406	0.023	0.504
**Child age**	0.785	0.108	0.187	**<0.001**	0.465	0.161	0.107	**0.004**
**Child weight status ^1^** (*Healthy weight*)								
Underweight	0.179	2.275	0.002	0.937	5.671	3.571	0.054	0.113
Overweight	−0.855	1.347	−0.015	0.526	0.453	1.941	0.008	0.816
Obesity	2.547	1.928	0.032	0.187	1.557	3.053	0.018	0.610
**Parent-proxy weight status ^1^** (*Healthy weight*)								
Underweight	−6.027	4.499	−0.032	0.181	6.220	8.130	0.026	0.445
Overweight	−1.576	1.160	−0.036	0.174	3.083	1.686	0.069	0.068
Obesity	1.025	1.221	0.022	0.401	5.307	1.842	0.110	**0.004**
**Parent-proxy education level** (*Tertiary*)								
Diploma/Certificate	4.387	1.247	0.105	**<0.001**	−0.371	1.775	−0.009	0.835
High school or less	1.721	1.310	0.041	0.189	1.381	1.922	0.030	0.473
**Parent-proxy equivalized income (deciles)**	−0.283	0.201	−0.038	0.159	−0.372	0.303	−0.048	0.220
**Parent-proxy employment status** (*Employed*)								
Unemployed	−3.134	2.873	−0.027	0.276	1.740	5.561	0.011	0.754
Not in Labor Force	0.706	1.282	0.015	0.582	−2.757	2.135	−0.049	0.197
**Parent-proxy discretionary choices intake (%E)**	0.302	0.023	0.318	**<0.001**	0.282	0.034	0.289	**<0.001**

^1^ Weight status according to BMI (kg/m^2^) categories; Underweight <18.5 kg/m^2^, Healthy weight 18.5–24.99 kg/m^2^, Overweight 25–29.99 kg/m^2^, Obesity >30 kg/m^2^. ^2^ Linear regression analysis adjusted for child physical activity and sedentary behavior and parent-proxy sex. ^3^ Model fit statistics were appropriate for both weekday (F(16, 1523) = 17.971, *p* < 0.001; R square 0.159) and weekend (F(16, 760) = 8.013, *p* < 0.001; R square 0.144) models.

## Data Availability

Please contact the Australian Bureau of Statistics regarding access to the National Nutrition and Physical Activity Survey 2011–2012 in de-identified format.

## Supplementary Materials

The following are available online at https://www.mdpi.com/article/10.3390/nu13114128/s1, Table S1: Characteristics of weekday and weekend samples of children and adolescents and parent-proxy from the National Nutrition and Physical Activity Survey 2011–2012 ^1^, by child age subgroups.
