# Supplementary material for: Diet Quality of Australian Children and Adolescents on Weekdays versus Weekend Days: A Secondary Analysis of the National Nutrition and Physical Activity Survey 2011–2012
Source: Nutrients. 2021 Nov 17;13(11):4128. doi: 10.3390/nu13114128 (PMC8621206; doi:10.3390/nu13114128)
Supplement: Supplementary file 1 [file nutrients-13-04128-s001.zip › nutrients-1443088-supplementary.pdf]

Dimity et al. **Diet quality of Australian children and adolescents on weekdays versus weekend days: A secondary analysis of the National Nutrition and Physical Activity Survey 2011-12; *Nutrients*, 2021**

Correspondence: brittany.johnson@flinders.edu.au

Table S1: Characteristics of weekday and weekend samples of children and adolescents and parent-proxy from the National Nutrition and Physical Activity Survey 2011-2012[1], by child age subgroups

	**Pre-school (2-4yo)**					**Primary School (5-11yo)**					**Secondary school (12-17yo)**				
	**Weekday** *(Monday-Thursday)*		**Weekend** *(Friday-Sunday)*		**p-value[6]**	**Weekday** *(Monday-Thursday)*		**Weekend** *(Friday-Sunday)*		**p-value[6]**	**Weekday** *(Monday-Thursday)*		**Weekend** *(Friday-Sunday)*		**p-value[6]**
	**n**	**%**	**n**	**%**		**n**	**%**	**n**	**%**		**n**	**%**	**n**	**%**	
Child Characteristics (2-17yo)	*n=448*		*n=197*			*n=674*		*n=329*			*n=604*		*n=332*		
**Sex**					0.630					0.643					0.093
Male	212	47.3	98	49.7		348	51.6	164	49.8		295	48.8	182	54.8	
Female	236	52.7	99	50.3		326	48.4	165	50.2		309	51.2	150	45.2	
**Age (years), mean (SD)**	3.0	0.8	2.9	0.8	0.630	8.0	2.0	8.0	0.2	0.820	14.6	1.7	14.5	1.7	0.399
**BMI (kg/m^2)[2]**					0.944					0.473					0.385
Underweight (<18.5)	26	7.4	11	6.7		31	5.7	10	3.5		18	3.6	11	3.9	
Healthy weight (18.5-24.99)	250	71.0	117	71.8		361	66.9	199	70.3		336	67.1	192	67.8	
Overweight (25-29.99)	56	15.9	24	14.7		101	18.7	53	18.7		100	20.0	63	22.3	
Obesity (>30)	20	5.7	11	6.7		47	8.7	21	7.4		47	9.4	17	6.0	
**Days met physical activity and screen-based recommendations, mean (SD)[3]**	3.7	2.6	3.9	2.6	0.260	3.8	2.3	3.8	2.3	0.854	1.8	1.9	2.2	2.1	0.022
Parent-proxy Characteristics (18+yo)	*n=448*		*n=197*			*n=674*		*n=329*			*n=604*		*n=332*		
**Sex**					0.112					0.012					0.164
Male	182	40.6	94	47.7		254	37.7	152	46.2		236	39.1	146	44.0	
Female	266	59.4	103	52.3		420	62.3	177	53.8		368	60.9	186	56.0	

Correspondence: brittany.johnson@flinders.edu.au

	**Pre-school (2-4yo)**					**Primary School (5-11yo)**					**Secondary school (12-17yo)**				
	**Weekday** *(Monday-Thursday)*		**Weekend** *(Friday-Sunday)*		**p-value[6]**	**Weekday** *(Monday-Thursday)*		**Weekend** *(Friday-Sunday)*		**p-value[6]**	**Weekday** *(Monday-Thursday)*		**Weekend** *(Friday-Sunday)*		**p-value[6]**
	**n**	**%**	**n**	**%**		**n**	**%**	**n**	**%**		**n**	**%**	**n**	**%**	
**Age (years), mean (SD)**	34.4	7.9	35.5	7.4	0.129	40.0	8.4	39.9	7.1	0.665	43.2	11.1	42.3	9.8	0.214
**BMI (kg/m²)[2]**					0.748					0.875					0.835
Underweight (<18.5)	9	2.4	3	1.7		5	0.9	2	0.7		5	1.0	5	1.7	
Healthy weight (18.5-24.99)	148	39.3	69	38.1		225	40.0	110	37.7		174	34.3	101	34.1	
Overweight (25-29.99)	128	34.0	69	38.1		185	32.9	103	35.3		166	32.7	99	33.4	
Obesity (>30)	92	24.4	40	22.1		147	26.2	77	26.4		162	32.0	91	30.7	
**Education Level**					0.195					0.140					0.444
Tertiary	149	33.3	80	40.6		193	28.6	110	33.4		146	24.2	78	23.5	
Diploma/Certificate	172	38.4	66	33.5		248	36.8	124	37.7		212	35.1	130	39.2	
High school or less	127	28.3	51	25.9		233	34.6	95	28.9		246	40.7	124	37.3	
**Employment Status**					0.239					**0.005**					0.055
Employed	309	69	146	74.1		500	74.2	274	83.3		470	77.8	280	84.3	
Unemployed	12	2.7	2	1.0		16	2.4	6	1.8		21	3.5	9	2.7	
Not in Labour Force	127	28.3	49	24.9		158	23.4	49	14.9		113	18.7	43	13.0	
**Household Income (deciles)[4], mean (SD)**	5.4	2.7	5.8	2.5	0.075	5.2	2.7	5.8	2.6	**0.001**	5.5	2.6	5.8	2.8	0.119
**SEIFA[5]**					0.729					0.128					0.337
Lowest quintile	86	19.2	29	14.7		116	17.2	61	18.5		103	17.1	44	13.3	
Second quintile	87	19.4	42	21.3		119	17.7	55	16.7		111	18.4	61	18.4	
Third quintile	92	20.5	41	20.8		142	21.1	49	14.9		135	22.4	68	20.5	
Fourth quintile	85	19.0	38	19.3		132	19.6	66	20.1		102	16.9	70	21.1	
Highest quintile	98	21.9	47	23.9		165	24.5	98	29.8		153	25.3	89	26.8	

[1] All results presented as N (%) unless reported otherwise

Correspondence: brittany.johnson@flinders.edu.au

[2] BMI, Body mass index categories; Underweight <18.5kg/m2; Normal weight 18.5-24.99kg/m2; Overweight 25-29.99kg/m2; Obesity >30kg/m2; missing data n=130 pre-school children, n=180 primary school children, n=152 secondary school children, n=87 preschool parent-proxy, n=149 primary school parent-proxy, n=133 secondary school parent-proxy

[3] Number of days met physical activity and screen-based recommendations in 7 days prior to interview, missing data n=26 pre-school children, n=11 primary school children, n=13 secondary school children

[4] Gross weekly equivalised cash income of household; 1st decile <$333, 2nd decile $333-$398, 3rd decile $399-$502, 4th decile $503-$638, 5th decile $639-$795, 6th decile $796-$958, 7th decile $959-$1151, 8th decile $1152-1437, 9th decile $1438-$1917, 10th decile >$1918, missing data n=33 preschool parent-proxy, n=67 primary school parent-proxy, n=122 secondary school parent-proxy

[5] SEIFA, Socio-Economic Indexes for Areas Index of Relative Socio-Economic Disadvantage 2011 – low index score indicated relatively greater disadvantage, high index score indicated a relative lack of disadvantage

[6] Differences between weekday and weekend samples tested using chi square for categorical variables (sex, BMI, education level, employment status, and SEIFA) and independent samples t-test or Mann Whitney U test for continuous variables (household income deciles; age and physical activity and screen-based recommendations, respectively)
